# Genome-Wide Association Analysis and Genomic Selection for Growth Traits in Grass Carp (*Ctenopharyngodon idella*)

**DOI:** 10.3390/ani15131888

**Published:** 2025-06-26

**Authors:** Yuxuan Chen, Qiaozhen Yu, Wenyao Lv, Tao Sheng, Lang Gui, Junqiang Qiu, Xiaoyan Xu, Jiale Li

**Affiliations:** 1 Key Laboratory of Freshwater Aquatic Genetic Resources, Ministry of Agriculture and Rural Affairs, Shanghai Ocean University, Shanghai 201306, China; 2 Key Laboratory of Integrated Rice-Fish Farming, Ministry of Agriculture and Rural Affairs, Shanghai Ocean University, Shanghai 201306, China; 3 Key Laboratory of Exploration and Utilization of Aquatic Genetic Resources, Ministry of Education, Shanghai Ocean University, Shanghai 266400, China; 4 Shanghai Engineering Research Center of Aquaculture, Shanghai Ocean University, Shanghai 201306, China

**Keywords:** grass carp, growth, liquid array, GWAS, genomic selection

## Abstract

Grass carp (*Ctenopharyngodon idella*), a globally important aquaculture species, exhibits protein-dependent growth plasticity, requiring genetic improvement for sustainable production. This study integrates genome-wide association analysis (GWAS) and genomic selection (GS) to unravel the genetic architecture of four growth traits, body weight, body length, body height, and body depth, in grass carp fed with diets of varying protein levels (20%, 25%, 30%, and 35%). Using a 21K liquid SNP array, we identified 62,736 high-quality SNPs across 24 chromosomes, with 90 SNPs significantly associated with growth traits. Notably, three SNPs (SLG14_24417024, SLG14_24417039, SLG24_30276273) exhibited pleiotropic effects on multiple traits. Functional annotation of 276 candidate genes near significant SNPs revealed enrichment in keratinocyte development, septin cytoskeleton organization, and heat acclimation pathways. Genomic prediction achieved accuracies up to 0.79 for body weight traits using 1533 optimal markers. This study provides the first comprehensive SNP resource for grass carp growth traits with different dietary treatments, bridging GWAS and genomic prediction to accelerate marker-assisted selection. Our findings not only advance genetic breeding strategies but also inform protein diet optimization, minimizing economic and environmental costs in aquaculture.

## Simple Summary

This study integrates genome-wide association analysis (GWAS) and genomic selection (GS) to unravel the genetic architecture of four growth traits, body weight, body length, body height, and body depth, in grass carp fed with diets of varying protein levels (20%, 25%, 30%, and 35%). This study provides the first comprehensive SNP resource for grass carp growth traits with different dietary treatments, bridging GWAS and genomic prediction to accelerate marker-assisted selection.

## 1. Introduction

Grass carp (*Ctenopharyngodon idella*), the most prolific freshwater aquaculture species worldwide [1], contributes critically to global food security and economic sustainability. However, optimizing its growth performance remains a priority, as feed costs—particularly protein—account for over 60% of production expenses. While previous studies have documented variable protein requirements among fish species [2], few investigations have integrated nutritional factors with genetic analyses to inform breeding strategies. Given the high cost of protein and its pivotal role in growth, it is imperative to elucidate the genetic mechanisms underlying protein utilization in grass carp to develop cost-effective feeding strategies.

Growth traits in fish are polygenic and influenced by genotype–environment interactions [3]. Traditional selective breeding has improved growth rates in grass carp, yet progress is constrained by prolonged generation intervals and low heritability estimates. Genomic technologies, notably genome-wide association studies (GWASs) and genomic selection (GS), offer transformative potential by linking molecular markers to phenotypes. In aquaculture, GWAS has been widely used to identify genetic loci associated with selective traits in fish, such as Atlantic salmon (*Salmo salar*) [4], yellowtail kingfish (*Seriola lalandi*), catfish [5], and rainbow trout (*Oncorhynchus mykiss*) [6]. Consequently, integrating these genomic tools into breeding programs is essential for uncovering critical genetic determinants that improve growth performance.

Protein intake directly modulates growth-related pathways, yet the genetic mechanisms underlying protein utilization in grass carp remain poorly characterized. Although earlier studies defined optimal dietary protein levels (35–45%) for juvenile grass carp, these recommendations lack genetic validation [2]. Determining the optimal protein level in fish diets is complex, as it is strongly influenced by dietary composition and experimental conditions. Genomic selection has successfully been incorporated into a few aquaculture species, including Pacific white shrimp [7], salmonid species [8], and common carp [9]. However, genomic selection—a cornerstone of modern aquaculture breeding—has not been systematically applied to grass carp. In particular, there is a pressing need to decipher the genetic basis for grass carp’s adaptation to low-protein diets, which could lead to improved dietary formulations and enhanced growth efficiency.

This study aims to (1) identify SNPs and candidate genes associated with growth traits in grass carp fed isoenergetic diets with graded protein levels (20–35%), and (2) evaluate genomic prediction models for breeding optimization. Using a novel 21K liquid SNP array, we performed GWAS on 928 individuals, followed by functional annotation and genomic selection via GMStool [10]. Our work provides the first high-resolution SNP resource for grass carp growth traits, integrating dietary and genomic data to advance precision aquaculture.

## 2. Material and Methods

### 2.1. Ethics Statement

All experiments performed on animals in this study were carried out according to the ethical guidelines of Shanghai Ocean University (Shanghai, China) on the care and use of experimental animals (approval number SHOU-DW-2018-026).

### 2.2. Sample Preparation for GWAS

Nine hundred and twenty-eight experimental grass carp came from Suzhou City, Jiangsu Province. Four types of protein feed were fed to the experimental grass carp, which contained four protein levels (20%, 25%, 30%, and 35%). Among them, 249 grass carp were fed with 20% protein feed, 153 grass carp were fed with 25% protein feed, 270 grass carp were fed with 30% protein feed, and 256 grass carp were fed with 35% protein feed. The experimental diet was fed twice per day, with 300 g provided each time. After feeding for three months, the 928 grass carp were collected for this study. Four growth-related traits including body weight (BW), body length (BL), body height (BH), and body depth (BD) were measured. The caudal fin of each individual was collected and provisionally kept in 100% absolute ethyl alcohol. Absolute ethyl alcohol was removed and saved at −80 °C for DNA extraction. The magnetic bead method was used for sample DNA extraction. A Qubit fluorescence quantitative analyzer was used to detect the concentration of DNA samples. The integrity of DNA samples was assessed by 1% agarose gel electrophoresis, and the qualified samples were used for library preparation.

### 2.3. SNP Array Genotyping

Genotyping of grass carp samples was performed using a 21K liquid array of grass carp on the DNBSEQ-T7 platform. Using PLINK v1.9 software to remove low-quality SNPs from the original data, the filtering parameters were “geno 0.1 -maf 0.05 -HWE 1 × 10^−6^” [11]. Burrows-Wheeler Aligner (BWA) software (MEM) was used to map clean reads to the grass carp reference genome. Finally, using GATK to call the variant [12], 928 grass carp retained a total of 62,736 SNPs.

### 2.4. Genome-Wide Association Study

Before GWAS analysis, principal component analysis (PCA) was implemented to assess the population structure [13]. A phylogenetic tree was constructed and visualized using ggplot2. This study used four models, emmax, emmaxQ, glm, and mlm, to conduct GWAS analysis on four growth traits of grass carp, and finally used the general linear model (GLM) of Tassel software (4.0). Furthermore, the Q matrix in the group structure analysis was taken as a covariate to correct the influence of the group structure. Bonferroni correction [14] was applied to determine the genome-wide significance threshold by calculating the formula *p* value = 0.05/N, where N represents the total number of markers used for association all drawn with R. The phenotypic variance explained (PVE) was calculated using Tassel software (4.0).

GMStool performs genome prediction based on genome-wide association studies (GWASs). GMStool heuristically searches for optimal markers using statistical and machine/deep learning models and presents the best prediction model with the optimal marker set [10]. Prediction modeling for each model was conducted by repeating p times (default 50), and in each p, 50 individuals were randomly selected from the training set obtained in the label selection stage as the validation set. Among all p modeling iterations of each model, the model with the highest correlation on the validation set was regarded as the final prediction model.

### 2.5. Candidate Gene Acquisition and Functional Annotation

The reference genome of grass carp (unpublished data) was applied to search for the candidate genes located in or near the significantly and suggestively associated SNPs (within 100 kb up- and downstream of the SNPs). The R package ClusterProfiler (ver. 4.12.0) was used for the GO and KEGG analysis of candidate genes, and the R package ggplot2 was used for visualization.

## 3. Results

### 3.1. Statistics of Growth Traits

**Figure 1:**
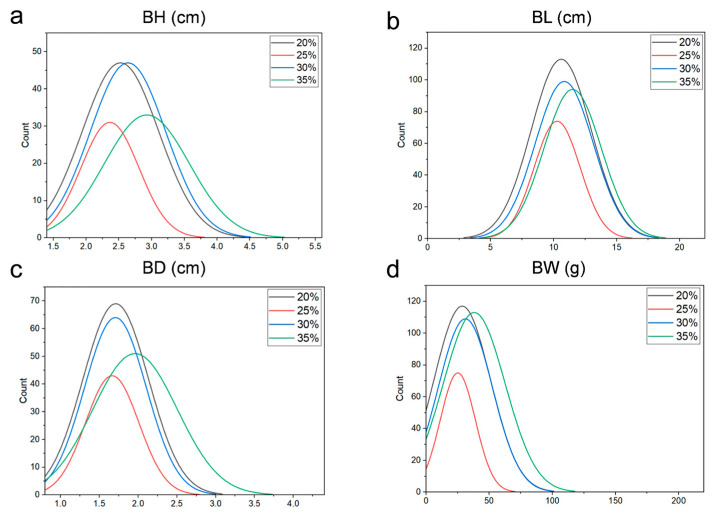
Frequency histogram of growth traits of grass carp on different protein feeds. (**a**) Body height (BH). (**b**) Body length (BL). (**c**) Body depth (BD). (**d**) Body weight (BW).

**Figure 2:**
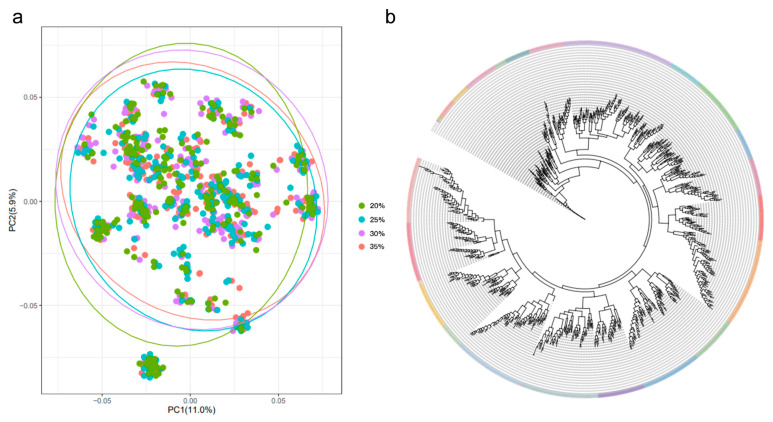
Results of population structure analysis for the grass carp experimental population. (**a**) Principal component analysis of the experimental population. (**b**) Cluster analysis of genotypes in the experimental groups.

Feeding grass carp with increasing dietary protein (20% to 35%) significantly enhanced growth traits (Figure 1). The protein 35% (P35) group exhibited the highest mean values for body weight (38.08 ± 24.43 g), body length (11.51 ± 2.27 cm), body height (2.93 ± 0.65 cm), and body depth (1.96 ± 0.54 cm), whereas the protein 20% (P20) group showed the lowest performance (BW: 28.60 ± 22.32 g; BL: 10.60 ± 2.38 cm; BH: 2.52 ± 0.59 cm; BD: 1.71 ± 0.42 cm) (Table 1). Principal component analysis (PCA) revealed that the level of genetic differences was low among populations fed different protein diets (Figure 2), indicating that phenotypic variations were primarily driven by genetic factors rather than population structure.

### 3.2. Genome-Wide Association Analysis

**Figure 3:**
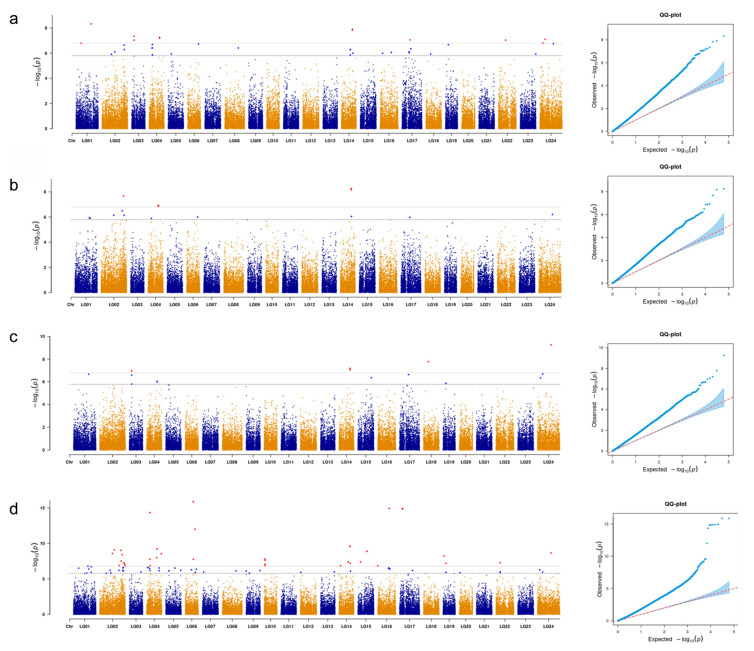
GWAS for growth-related traits in grass carp. (**a**–**d**) Manhattan and quantile–quantile (Q-Q) plots for body height ((**a**), BH), body length ((**b**), BL), body depth ((**c**), BD), and body weight ((**d**), BW), respectively. The dotted line represents the significance threshold.

Due to the lack of significant loci in the GWAS results of the emmax and emmaxQ models, this study adopted the GWAS results of the glm model. In order to reduce model errors and improve statistical efficiency, feed protein levels were used as covariates. A total of 90 SNPs were found to be significantly associated with the growth traits and were distributed across 14 chromosomes (Table S1). Specifically, 27 SNPs were associated with body height (3.03% of PVE), 11 with body length (2.91%), 76 with body weight (2.09%), and 11 with body depth (3.54%) (Figure 3). Notably, three SNPs, SLG14_24417024, SLG14_24417039, and SLG24_30276273, displayed pleiotropic effects, being significantly associated with multiple traits including body height, body length, and body weight.

### 3.3. Candidate Gene Prediction

**Figure 4:**
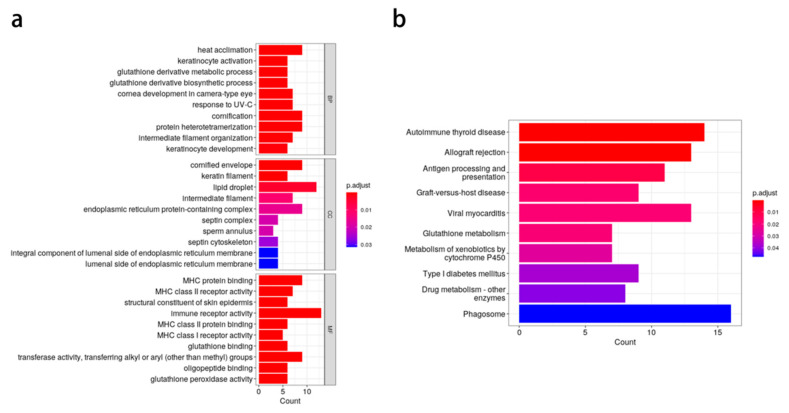
Functional annotation of candidate genes with growth-related SNP sites. (**a**) GO categories. (**b**) KEGG pathways. BP, biological process; CC, cell component; MF, molecular function.

We identified 276 candidate genes flanking the significant SNPs. Gene Ontology (GO) enrichment analysis of these genes revealed a total of 93 enriched GO terms, categorized into 53 biological processes, 12 cellular components, and 28 molecular functions (Figure 4). Notably, several GO terms related to keratinocyte development and septin cytoskeleton organization were significantly enriched. The KEGG pathway analysis indicated that the candidate genes were predominantly involved in pathways related to allogeneic rejection, antigen processing and presentation, and glutathione metabolism.

### 3.4. Genomic Prediction Within Population

For growth characteristics, including body height, body length, body width, and weight, we evaluated four GWAS models (emmax, emmaxQ, glm, mlm), two marker selection strategies (RRB and RRB_BTS), and four machine learning algorithms (RRB, RF, DNN, CNN). The genome prediction model achieved an optimal correlation of 0.62–0.79 between the observed and predicted phenotypes in four growth traits (BH, BL, BD, and BW) (Table 2). The RRB_BTS marker selection method based on the GWAS model of emmaxQ performed optimally, selecting 1571–1949 SNPs for body height, length, depth, and weight traits. Body weight traits showed the highest prediction accuracy (correlation = 0.79) under the DNN model, emphasizing the utility of machine learning for complex trait improvement.

## 4. Discussion

### 4.1. Genetic Architecture of Protein-Dependent Growth Traits

In this study, we conducted a genome-wide association study (GWAS) on grass carp with diets containing different protein levels (20%, 25%, 30%, and 35%) using a 21K liquid-phase SNP array. Four models (glm, mlm, emmax, emmaxQ) were used for GWAS analysis, while no significantly associated SNPs were found in emmax and emmaxQ. The results of principal component analysis showed that the genetic differences among the experimental groups were not obvious. When a mixed model is constructed using the kinship matrix, it might overfit the population structure. The glm model retains the efficacy of true associations using the Q matrix as a covariate. When the feed protein level is taken as a covariate, the glm model can utilize more abundant information, avoid over-simplification of the model, reduce model errors, and thereby improve the model’s ability to detect significant loci. Our GWAS identified 90 SNPs significantly associated with key growth traits, including body weight, body length, body height, and body depth, with several SNPs displaying pleiotropic effects. Notably, SNPs SLG14_24417024, SLG14_24417039, and SLG24_30276273 were associated with multiple traits, suggesting that these loci may play central roles in the regulation of growth. Both the SLG14_24417024 and SLG14_24417039 loci are located near the *SLC5A6* gene, and there is a connection between the *SLC5A6* gene and growth regulation. These two SNP loci may affect gene expression or function, and also influence traits such as weight, body length, and body height. They may be truly pleiotropic loci. However, SLG24_30276273 is located alone near the *LIG3* gene, making it difficult to determine whether it is a single gene with multiple effects or a multiple-gene linkage. More functional verifications are still needed to clarify its mechanism of action. Additionally, we referred to the report on the growth trait QTL of grass carp by Guo et al. and found that the position of its QTL was relatively far from the positions of SLG14_24417024, SLG14_24417039, and SLG24_30276273 in this study [15]. The presence of SNPs in both exonic and intergenic regions is indicative of the complex genetic architecture underlying these traits. GWAS analysis was conducted on the growth traits of black carp, and the most significant SNP, chr 05_ 42410277, was found to be significantly associated with body weight and length [16]. Yang et al. conducted GWAS on the growth traits of brown-marbled grouper, and the results showed that there were five SNPs significantly associated with growth traits, among which SNP locus 23:29601315 was significantly associated with body weight, length, height, and thickness. SNP 23:29601315 was validated. The C allele (18.3%) was superior and the A allele (81.7%) was inferior for growth traits. The C allele could be enriched in future selection of brown-marbled grouper based on genotypes at this locus [17]. In a GWAS analysis of grass carp, multiple SNPs were found to be significantly associated with two or three traits simultaneously [18]. Growth traits are controlled by multiple genes, and SNPs related to growth are dispersed on multiple chromosomes, possibly due to the joint influence of different genes or chromosomal regions on fish growth traits. SNP overlap association contributes to the enrichment of advantageous variations in growth and the reduction in disadvantageous variations.

Candidate gene analysis, performed within a ±100 kb window of significant SNPs, yielded 276 genes. GO and KEGG pathway enrichment analyses revealed significant involvement of these genes in processes such as keratinocyte development, septin cytoskeleton organization, and heat acclimation. For instance, the proximity of SLG14_24417024 and SLG14_24417039 to the *SLC5A6* gene [19], known for its role in biotin and pantothenic acid transport [20,21], suggests a possible link between nutrient absorption and growth regulation. An intestine-specific (conditional) *SLC5A6* knockout (KO) mouse showed inhibited intestinal biotin uptake, exhibiting slow growth and impaired bone development. Meanwhile, the homologous gene *SLC5A6a* of *SLC5A6* was localized in a genome-wide association analysis of head size and shape in bighead carp [22]. Similarly, the identification of an SNP within the *LIG3* gene [23], which plays a crucial role in DNA repair, points to the importance of genomic integrity in maintaining optimal growth performance. The destruction of *LIG3* in zebrafish leads to changes in the brain and impaired intestinal transport. Therefore, mutations in the *LIG3* gene may lead to mitochondrial diseases characterized by major intestinal motility disorders, brain diseases, and neuromuscular abnormalities [22]. The clathrin-mediated endocytosis encoded by the clathrin interacting protein 1 (*CLINT1*) gene is an important mechanism for the cellular uptake of macromolecules such as proteins [23]. Endocytosis may be involved in protein absorption and transport in the intestine or other tissues. *CLINT1* plays a significant role in epidermal development and inflammation. Studies have shown that zebrafish lacking this protein can produce a phenotype similar to that of human psoriasis [23]. *TMPRSS9* belongs to the transmembrane serine protease family. Proteases are a class of enzymes that hydrolyze proteins and participate in food digestion through non-specific protein hydrolysis, and in more diverse physiological processes [24]. These findings are consistent with previous reports on genetic influences on fish growth traits [25] and underscore the potential of using these markers in molecular-assisted breeding programs.

### 4.2. Advancing Precision Breeding Through Genomic Prediction

GS analyses based on GWAS-derived markers demonstrate that integrating multiple prediction models—such as RRB, RF, DNN, and CNN—can substantially enhance the accuracy of estimating breeding values in grass carp. These findings suggest that the complex genetic architecture of growth traits benefits from a multi-model approach, as no single model consistently provides the optimal prediction for all phenotypes [26]. This multi-model strategy allows us to capture both additive and non-additive genetic effects, thereby providing a more robust framework for genomic prediction in aquaculture species.

Notably, the high genomic prediction accuracy (r = 0.79 for body weight) achieved with a set of 1533 SNPs underscores the feasibility of marker-assisted selection in grass carp. Compared to traditional best linear unbiased prediction (BLUP) methods—which generally yield lower predictive accuracies—the multi-trait CNN model employed in our study significantly improves selection efficiency. Genome prediction for growth traits in juvenile farmed Atlantic salmon (Salmo salar) using high-density SNP arrays revealed that genomic best linear unbiased prediction (GBLUP) produced more accurate breeding value estimates than pedigree-based BLUP (PBLUP) relationship matrices (accuracy ~ 0.7 and 0.58, respectively) [27]. GS analysis was conducted on Edwardsiella tarda in Japanese flounder (*Paralichthys olivaceus*), and it was found that compared to pedigree-based prediction (ABLUP), the three genomic methods (ssGBLUP, WssGBLUP, BayesB) evaluated showed an improvement in prediction accuracy of at least 7.7% [28]. Genomic selection (GS) analysis was conducted to evaluate resistance to sea lice (Caligus rogercresseyi) in Atlantic salmon (Salmo salar). The accuracy of breeding value predictions obtained using pedigree-based best linear unbiased prediction (P-BLUP) was compared with that of several genomic prediction methods: genomic BLUP (G-BLUP), Bayesian Lasso, and Bayes C. The results indicated that both the Bayes C (0.50) and G-BLUP (0.50) methods can predict breeding values with higher accuracies than pedigree-based BLUP (0.41) [29]. This enhanced accuracy supports the application of genomic selection to accelerate genetic gain, ultimately contributing to more efficient breeding strategies and improved economic sustainability in aquaculture.

## 5. Conclusions

This study combined growth experiments, GWAS, and genomic selection to reveal the genetic basis of key growth traits in grass carp under varying dietary protein levels. We identified 90 significant SNPs across 24 chromosomes and 276 candidate genes involved in processes such as keratinocyte development and septin cytoskeleton organization. Notably, loci SLG14_24417024, SLG14_24417039, and SLG24_30276273 exhibited pleiotropic effects, underscoring their potential for marker-assisted selection. Advanced genomic prediction models achieved high accuracy (r = 0.79 for body weight using 1533 SNPs), demonstrating the feasibility of accelerating genetic gains in grass carp. Overall, our integrative approach not only advances the understanding of the genetic architecture underlying growth traits in grass carp but also paves the way for more sustainable and economically viable breeding strategies in the aquaculture industry.

## Figures and Tables

**Table 1:** Growth traits of grass carp fed with different protein diets. The unit of measurement for body height, length, and depth is cm, and the unit of measurement for body weight is g.

	20% Protein Feed				25% Protein Feed				30% Protein Feed				35% Protein Feed			
	BL (cm)	BH (cm)	BD (cm)	BW (g)	BL (cm)	BH (cm)	BD (cm)	BW (g)	BL (cm)	BH (cm)	BD (cm)	BW (g)	BL (cm)	BH (cm)	BW (cm)	BD (g)
average value	10.60	2.52	1.71	28.60	10.27	2.36	1.67	25.11	18.84	2.64	1.71	31.10	11.51	2.93	1.96	30.08
max value	19.61	4.61	3.24	153.94	15.67	3.64	2.74	73.7	19.23	4.64	3.41	141.65	21.40	5.37	4.24	207.97
min value	7.11	1.61	1.00	7.04	6.19	1.70	1.08	8.68	1.41	1.70	1.06	9.40	7.37	1.76	1.09	9.17

**Table 2:** Whole marker set and correlation of GS.

Phenotype	GWAS	Method	Select Markers	RRB (Train/Val./Test)	RF (Train/Val./Test)	DNN (Train/Val./Test)	CNN (Train/Val./Test)
BH	glm	RRB	2138	0.96/0.86/0.51	0.99/0.45/0.57	0.00/0.14/0.00	0.85/0.47/0.39
		RRB_BTS	2195	0.96/0.86/0.51	0.99/0.47/0.60	0.08/0.27/0.44	0.85/0.53/0.53
	mlm	RRB	1529	0.95/0.87/0.65	0.99/0.52/0.63	0.02/0.10/0.27	0.87/0.64/0.67
		RRB_BTS	1572	0.95/0.87/0.65	0.99/0.54/0.63	0.07/0.06/0.10	0.87/0.61/0.65
	emmax	RRB	1678	0.95/0.88/0.70	0.99/0.50/0.58	0.05/0.06/0.00	0.86/0.63/0.61
		RRB_BTS	1701	0.95/0.87/0.70	0.99/0.51/0.58	0.00/0.12/−0.26	0.85/0.67/0.55
	emmaxQ	RRB	1579	0.94/0.86/0.71	0.99/0.50/0.55	0.12/0.25/0.11	0.87/0.64/0.59
		RRB_BTS	1636	0.94/0.86/0.71	0.99/0.49/0.57	0.00/0.10/−0.12	0.87/0.68/0.66
BL	glm	RRB	2094	0.95/0.85/0.50	0.98/0.45/0.65	0.90/0.57/0.35	0.88/0.57/0.52
		RRB_BTS	2154	0.94/0.85/0.51	0.98/0.48/0.66	0.86/0.56/0.41	0.92/0.54/0.42
	mlm	RRB	1478	0.94/0.87/0.71	0.99/0.54/0.70	0.89/0.70/0.59	0.93/0.66/0.66
		RRB_BTS	1515	0.94/0.87/0.71	0.99/0.50/0.71	0.87/0.69/0.71	0.93/0.66/0.64
	emmax	RRB	1758	0.950.88/0.72	0.99/0.48/0.66	0.88/0.69/0.59	0.92/0.60/0.55
		RRB_BTS	1780	0.95/0.87/0.71	0.99/0.47/0.66	0.89/0.71/0.65	0.91/0.65/0.61
	emmaxQ	RRB	1687	0.94/0.87/0.72	0.99/0.46/0.68	0.89/0.70/0.60	0.92/0.62/0.68
		RRB_BTS	1719	0.94/0.87/0.72	0.99/0.45/0.69	0.90/0.66/0.64	0.91/0.62/0.56
BD	glm	RRB	2070	0.96/0.86/0.34	0.98/0.49/0.49	0.15/0.29/0.21	0.72/0.47/0.30
		RRB_BTS	2134	0.96/0.85/0.34	0.98/0.50/0.48	0.12/0.15/−0.01	0.78/0.46/0.35
	mlm	RRB	1640	0.94/0.84/0.59	0.99/0.53/0.55	0.09/0.11/0.15	0.79/0.58/0.39
		RRB_BTS	1649	0.94/0.83/0.60	0.99/0.53/0.56	−0.01/0.10/0.17	0.80/0.58/0.53
	emmax	RRB	1880	0.95/0.85/0.62	0.98/0.47/0.50	0.10/0.18/−0.05	0.82/0.59/0.55
		RRB_BTS	1880	0.95/0.85/0.62	0.98/0.47/0.50	0.10/0.18/−0.04	0.82/0.59/0.55
	emmaxQ	RRB	1903	0.94/0.85/0.60	0.99/0.48/0.51	0.03/0.18/0.18	0.80/0.62/0.57
		RRB_BTS	1949	0.94/0.85/0.60	0.99/0.49/0.51	0.03/0.10/0.35	0.84/0.57/0.49
BW	glm	RRB	NA	NA	NA	NA	NA
		RRB_BTS	NA	NA	NA	NA	NA
	mlm	RRB	NA	NA	NA	NA	NA
		RRB_BTS	NA	NA	NA	NA	NA
	emmax	RRB	1524	0.94/0.86/0.74	0.98/0.48/0.70	0.93/0.78/0.72	0.89/0.68/0.64
		RRB_BTS	1554	0.94/0.86/0.74	0.98/0.48/0.70	0.94/0.77/0.73	0.91/0.67/0.56
	emmaxQ	RRB	1533	0.94/0.86/0.76	0.98/0.47/0.71	0.94/0.78/**0.79**	0.89/0.68/0.68
		RRB_BTS	1571	0.94/0.86/0.76	0.98/0.48/0.71	0.94/0.77/0.76	0.93/0.68/0.61

Notes: Selection marker is the combination of SNP markers obtained from different selection methods as the optimal marker group. Val is validation set.

## Data Availability

None of the data was deposited in an official repository but is available from the authors upon request.

## Supplementary Materials

The following supporting information can be downloaded at https://www.mdpi.com/article/10.3390/ani15131888/s1, Table S1: SNP loci associated with growth traits.

## References

[1] Yu C., Tang H., Jiang Y., Lu H., Chen Q., Gui L., Qiu J., Xu X., Li J., Shen Y. (2024). Growth Performance and Selection Signatures Revealed by Whole-Genome Resequencing in Genetically Selected Grass Carp (*Ctenopharyngodon idella*). Aquaculture.

[2] Jin Y., Tian L., Xie S., Guo D., Yang H., Liang G., Liu Y. (2015). Interactions between Dietary Protein Levels, Growth Performance, Feed Utilization, Gene Expression and Metabolic Products in Juvenile Grass Carp (*Ctenopharyngodon idella*). Aquaculture.

[3] Korte A., Farlow A. (2013). The Advantages and Limitations of Trait Analysis with GWAS: A Review. Plant Methods.

[4] Sodeland M., Gaarder M., Moen T., Thomassen M., Kjøglum S., Kent M., Lien S. (2013). Genome-Wide Association Testing Reveals Quantitative Trait Loci for Fillet Texture and Fat Content in Atlantic Salmon. Aquaculture.

[5] Jin Y., Zhou T., Geng X., Liu S., Chen A., Yao J., Jiang C., Tan S., Su B., Liu Z. (2017). A Genome-Wide Association Study of Heat Stress-Associated SNPs in Catfish. Anim. Genet..

[6] Reis Neto R.V., Yoshida G.M., Lhorente J.P., Yáñez J.M. (2019). Genome-Wide Association Analysis for Body Weight Identifies Candidate Genes Related to Development and Metabolism in Rainbow Trout (*Oncorhynchus mykiss*). Mol. Genet. Genom..

[7] Castillo-Juárez H., Campos-Montes G.R., Caballero-Zamora A., Montaldo H.H. (2015). Genetic Improvement of Pacific White Shrimp [Penaeus (Litopenaeus) Vannamei]: Perspectives for Genomic Selection. Front. Genet..

[8] D’Agaro E., Favaro A., Matiussi S., Gibertoni P.P., Esposito S. (2021). Genomic Selection in Salmonids: New Discoveries and Future Perspectives. Aquacult. Int..

[9] Su S., Raouf B., He X., Cai N., Li X., Yu J., Li J., Yu F., Wang M., Tang Y. (2020). Genome Wide Analysis for Growth at Two Growth Stages in A New Fast-Growing Common Carp Strain (*Cyprinus carpio* L.). Sci. Rep..

[10] Jeong S., Kim J.-Y., Kim N. (2020). GMStool: GWAS-Based Marker Selection Tool for Genomic Prediction from Genomic Data. Sci. Rep..

[11] Purcell S., Neale B., Todd-Brown K., Thomas L., Ferreira M.A.R., Bender D., Maller J., Sklar P., de Bakker P.I.W., Daly M.J. (2007). PLINK: A Tool Set for Whole-Genome Association and Population-Based Linkage Analyses. Am. J. Hum. Genet..

[12] McKenna A., Hanna M., Banks E., Sivachenko A., Cibulskis K., Kernytsky A., Garimella K., Altshuler D., Gabriel S., Daly M. (2010). The Genome Analysis Toolkit: A MapReduce Framework for Analyzing next-Generation DNA Sequencing Data. Genome Res..

[13] Zhou Y., Fu H.-C., Wang Y.-Y., Huang H.-Z. (2022). Genome-Wide Association Study Reveals Growth-Related SNPs and Candidate Genes in Mandarin Fish (*Siniperca chuatsi*). Aquaculture.

[14] Bonferroni C. (1936). Teoria Statistica Delle Classi e Calcolo Delle Probabilita. Pubblicazioni del R Istituto Superiore di Scienze Economiche e Commericiali di Firenze.

[15] Guo J., Zhang M., Wang S., Xu X., Shen Y., Li J. (2022). A High-Density Genetic Linkage Map and QTL Mapping for Growth Related Traits in Grass Carp (*Ctenopharyngodon idella*). Aquaculture.

[16] Zhou S., Yang L., Li J., Shen Y. (2025). Genome-Wide Association Study for Growth Traits in Black Carp (*Mylopharyngodon piceus*). Aquaculture.

[17] Yang Y., Wu L., Wu X., Li B., Huang W., Weng Z., Lin Z., Song L., Guo Y., Meng Z. (2020). Identification of Candidate Growth-Related SNPs and Genes Using GWAS in Brown-Marbled Grouper (*Epinephelus fuscoguttatus*). Mar. Biotechnol..

[18] Zhang D.-Y., Luo L.-F., Wang Z.-Y., Yu Y., Nie C.-H., Guo X.-Z., Gao Z.-X. (2024). Identification of Novel SNPs and Candidate Genes Significantly Affecting Growth in Grass Carp (*Ctenopharyngodon idella*) through GWAS Analysis. Aquaculture.

[19] Chen G., Zhou Y., Yu X., Wang J., Luo W., Pang M., Tong J. (2022). Genome-Wide Association Study Reveals SNPs and Candidate Genes Related to Growth and Body Shape in Bighead Carp (*Hypophthalmichthys nobilis*). Mar. Biotechnol..

[20] Ghosal A., Lambrecht N., Subramanya S.B., Kapadia R., Said H.M. (2013). Conditional Knockout of the Slc5a6 Gene in Mouse Intestine Impairs Biotin Absorption. Am. J. Physiol. Gastrointest. Liver Physiol..

[21] Wang J., Yu X., Chen G., Zhang Y., Tong J. (2022). Molecular Characterization of *Slc5a6a* and Its Association with Growth and Body Conformation in Bighead Carp (*Hypophthalmichthys nobilis*). Aquac. Rep..

[22] Lee E.-R., Kim J.-Y., Kang Y.-J., Ahn J.-Y., Kim J.-H., Kim B.-W., Choi H.-Y., Jeong M.-Y., Cho S.-G. (2006). Interplay between PI3K/Akt and MAPK Signaling Pathways in DNA-Damaging Drug-Induced Apoptosis. Biochim. Biophys. Acta.

[23] Pimm J., McQuillin A., Thirumalai S., Lawrence J., Quested D., Bass N., Lamb G., Moorey H., Datta S.R., Kalsi G. (2005). The Epsin 4 Gene on Chromosome 5q, Which Encodes the Clathrin-Associated Protein Enthoprotin, Is Involved in the Genetic Susceptibility to Schizophrenia. Am. J. Human Genet..

[24] Guipponi M., Toh M.-Y., Tan J., Park D., Hanson K., Ballana E., Kwong D., Cannon P.Z.F., Wu Q., Gout A. (2008). An Integrated Genetic and Functional Analysis of the Role of Type II Transmembrane Serine Proteases (TMPRSSs) in Hearing Loss. Human Mutat..

[25] Hao Y., Jia X., Yuan L., Liu Y., Gui L., Shen Y., Li J., Xu X. (2023). Genome-Wide Association Study Reveals Growth-Related SNPs and Candidate Genes in Grass Carp (*Ctenopharyngodon idella*). Aquaculture.

[26] Azodi C.B., Bolger E., McCarren A., Roantree M., de los Campos G., Shiu S.-H. (2019). Benchmarking Parametric and Machine Learning Models for Genomic Prediction of Complex Traits. G3 Genes Genomes Genet..

[27] Tsai H.-Y., Hamilton A., Tinch A.E., Guy D.R., Gharbi K., Stear M.J., Matika O., Bishop S.C., Houston R.D. (2015). Genome Wide Association and Genomic Prediction for Growth Traits in Juvenile Farmed Atlantic Salmon Using a High Density SNP Array. BMC Genom..

[28] Lu S., Liu Y., Yu X., Li Y., Yang Y., Wei M., Zhou Q., Wang J., Zhang Y., Zheng W. (2020). Prediction of Genomic Breeding Values Based on Pre-Selected SNPs Using ssGBLUP, WssGBLUP and BayesB for Edwardsiellosis Resistance in Japanese Flounder. Genet. Sel. Evol..

[29] Correa K., Bangera R., Figueroa R., Lhorente J.P., Yáñez J.M. (2017). The Use of Genomic Information Increases the Accuracy of Breeding Value Predictions for Sea Louse (*Caligus rogercresseyi*) Resistance in Atlantic Salmon (*Salmo salar*). Genet. Sel. Evol..

